# A Comparison of Conserved Features in the Human Coronavirus Family Shows That Studies of Viruses Less Pathogenic than SARS-CoV-2, Such as HCoV-OC43, Are Good Model Systems for Elucidating Basic Mechanisms of Infection and Replication in Standard Laboratories

**DOI:** 10.3390/v17020256

**Published:** 2025-02-13

**Authors:** Audrey L. Heffner, Tracey A. Rouault

**Affiliations:** 1 Molecular Medicine Branch, *Eunice Kennedy Shriver* National Institute of Child Health and Human Development, Bethesda, MD 20892, USA; 2 Department of Biology, Johns Hopkins University, Baltimore, MD 21218, USA

**Keywords:** endemic coronaviruses, cytopathic effect, HCoV-OC43, HCoV-229E, HCoV-NL63, HCoV-HKU1

## Abstract

In 2021, at the height of the COVID-19 pandemic, coronavirus research spiked, with over 83,000 original research articles related to the word “coronavirus” added to the online resource *PubMed*. Just 2 years later, in 2023, only 30,900 original research articles related to the word “coronavirus” were added. While, irrefutably, the funding of coronavirus research drastically decreased, a possible explanation for the decrease in interest in coronavirus research is that projects on SARS-CoV-2, the causative agent of COVID-19, halted due to the challenge of establishing a good cellular or animal model system. Most laboratories do not have the capabilities to culture SARS-CoV-2 ‘in house’ as this requires a Biosafety Level (BSL) 3 laboratory. Until recently, BSL 2 laboratory research on endemic coronaviruses was arduous due to the low cytopathic effect in isolated cell culture infection models and the lack of means to quantify viral loads. The purpose of this review article is to compare the human coronaviruses and provide an assessment of the latest techniques that use the endemic coronaviruses—HCoV-229E, HCoV-OC43, HCoV-NL63, and HCoV-HKU1—as lower-biosafety-risk models for the more pathogenic coronaviruses—SARS-CoV-2, SARS-CoV, and MERS-CoV.

## 1. Introduction

Coronaviruses are zoonotic pathogens, with many having the potential to infect humans and cause severe disease, posing a concerning, continual global threat to humanity [1,2]. In recent years, much attention has been given to human coronaviruses; however, this was not the case in the years immediately following their discovery. The first two human endemic seasonal common cold coronaviruses, HCoV-229E and HCoV-OC43, were identified in the 1960s and determined to be distinct from each other when the serologic antibodies of an individual infected with one coronavirus did not react with the spike protein of the other [3,4,5]. Before the initial outbreak of a mysterious and fatal disease in China in 2003—severe acute respiratory syndrome, now known to be caused by a human coronavirus, SARS-CoV [6,7,8]—coronaviruses were thought to be inconsequential and were only associated with the common cold and upper respiratory illness in children [3,4]. Before SARS-CoV emerged, little progress had been made in understanding the epidemiology of human endemic coronaviruses, and no attempts had been made to develop a vaccine or therapeutic options [9].

The outbreak of SARS-CoV heightened scientific interest in coronaviruses, leading to the discovery of two additional endemic coronaviruses, namely, HCoV-HKU1 and HCoV-NL63 [10,11,12,13]. However, challenges in culturing endemic coronaviruses in isolated cell lines and a lack of non-cytopathic viral quantification methods led to research stagnation regarding these coronaviruses [14]. The 2012 outbreak of what is now known as the Middle East Respiratory Syndrome coronavirus (MERS-CoV), just 10 years after the SARS-CoV outbreak, raised concerns among scientists about the potential of coronaviruses to jump species and spread among the human population rapidly [15,16]. Less than a decade later, the world came to a standstill with the COVID-19 pandemic, caused by the coronavirus SARS-CoV-2 in 2019 [17,18].

The scientific community made significant advances during the pandemic, developing and employing novel RNA-based vaccines, antibodies, and protease-targeting antivirals against SARS-CoV-2 [19,20,21,22]. These efforts significantly reduced the impact of COVID-19, although the impact of the pandemic was considerable. With these resources now available, researchers must prepare for the ever-evolving variants of SARS-CoV-2 and the possibility of novel coronaviruses expanding to the human population from animal carriers.

One research strategy that has gained popularity in recent years is using less pathogenic endemic coronaviruses, as safer and more approachable alternatives, to discover antivirals and understand coronavirus biology [14,23,24,25]. In this review, we compare human endemic coronaviruses to their more pathogenic counterparts and outline recent advances in the culturing of endemic coronaviruses (HCoV-229E, HCoV-OC43, HCoV-NL63, and HCoV-HKU1) as safer replacements for the more pathogenic coronaviruses (SARS-CoV, MERS-CoV, SARS-CoV-2) used in discovery studies. The increased attention given to endemic coronaviruses is well deserved. It will likely lead to breakthroughs in the development of antivirals, insights into host and viral biology, and a better understanding of how and when viruses become endemic—an essential question that will inform current policies and the treatment of SARS-CoV-2 worldwide.

## 2. Coronavirus Infection Basics

### 2.1. The Components of a Coronavirus Virion

**Figure 1:**
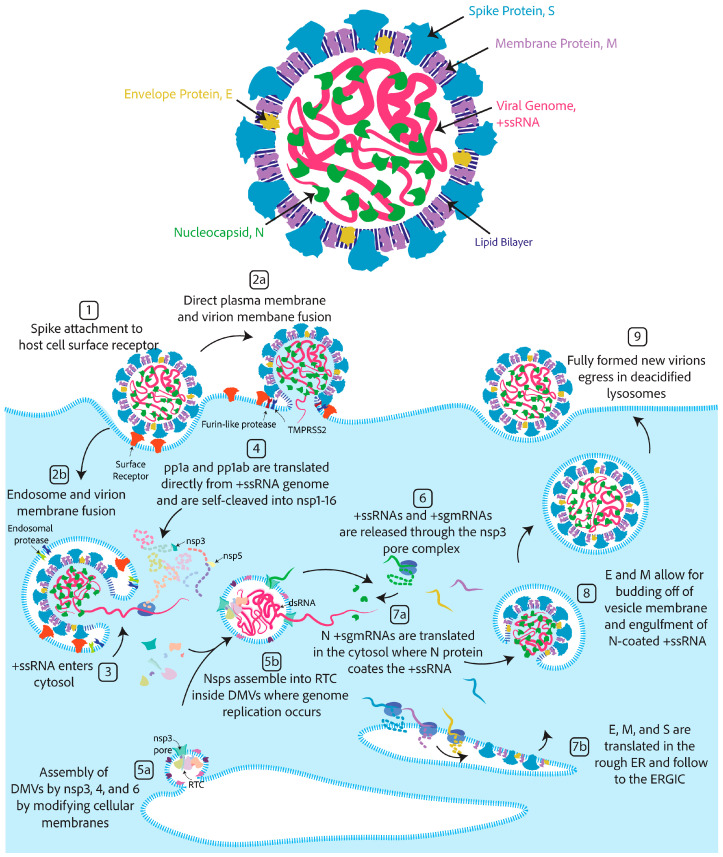
Components of the coronavirus virion and key stages of the coronavirus infection cycle. Top: the coronavirus virion components. Bottom: the coronavirus infection cycle. (1) Spike attachment to a cell surface exposed adhesion factor. For betacoronaviruses, a furin protease cleaves the spike in the S1/S2 site. The spike is additionally cleaved in the S2’ site by either a cell surface protease like TMPRSS2 (2a) or an endosomal protease due to the endosomal pathway being triggered (2b). After the cleavage of both sites, the spike is activated for membrane fusion. (3) The virion contents spill into the cell cytosol; concomitantly, the nucleocapsid releases the single-strand RNA genome. (4) Translation by cellular ribosomes of the 5′ end open reading frames ORF1a and ORF1ab. The self-cleavage of the polypeptides into 16 nonstructural proteins, called nsp1–16, by the protease domain of nsp3, and nsp5. Nsp3, nsp4, and nsp6 are responsible for the formation of double-membrane vesicles (DMVs) or replication organelles that house the replicating genome in order to protect the double-stranded RNA intermediates from host cell pAMPs (5a). Several nsps form the replication and transcription complex (5b). Genome replication by continuous RNA synthesis and transcription of 3′ end genes by discontinuous RNA synthesis occurs in double-membrane vesicles. (6) Resulting +ssRNAs and +sgmRNAs are released into the cytosol through the DMV pore complex formed by nsp3. (7) The translation of S, E, and M into the rough ER, populating the ER membrane and flowing throughout the ER–Golgi, and the translation of N into the cytosol, which encapsulates free +ssRNA genomes. (8) The E and M proteins are responsible for the virion budding off cellular membranes and the engulfment of N-coated +ssRNA. (9) Newly formed virions egress in deacidified lysosomes in a non-lytic manner.

The virion is the infectious unit capable of attaching to and entering susceptible host cells. It houses the genetic material encoding the proteins necessary to mass produce more of itself [26]. The coronavirus virion (Figure 1) is a spherical double-membraned or enveloped spherical structure that is approximately 80–120 nanometers in diameter [26,27].

Each coronavirus virion has three membrane proteins: the spike (S), the membrane (M), and the envelope protein (E) [26]. The membrane protein is the most abundant, followed by the spike protein and the envelope protein [26]. Both the membrane and envelope proteins are separately capable of forming spherical structures when expressed in noninfected cells, suggesting they are involved in regulating the size and shape of the virion [28]. The spike is responsible for attachment to host cell receptors and the entry of the genetic material into the host cell cytoplasm [29,30,31]. A subgenus of betacoronavirus known as embecovirus has one additional virion structural protein, the hemagglutinin-esterase [26]. The hemagglutinin-esterase recognizes cell surface receptors and subsequently destroys them [32]. Its function is similar to that of the influenza hemagglutinin-esterase-fusion protein of influenza C/D, which combines the functions of receptor binding and receptor destruction [32]. The receptor-destroying activity has dual functions: (1) deterring the re-infection of an already infected cell and (2) mitigating attachment to immune decoys of non-cell surface-attached receptors free floating in mucosal fluids [33].

Inside the coronavirus virion is a single strand of RNA coated in a nucleocapsid protein (N) that coils the RNA [26]. Coronavirus RNA has coding potential, thus making it so-called positive-sense single-stranded RNA (+ssRNA) [26]. Coronavirus RNA is ~30 kilobases, which is abnormally long compared to the average RNA viral genome of ~9 kilobases [34].

### 2.2. Coronavirus Lifecycle in Brief

**Figure 2:**
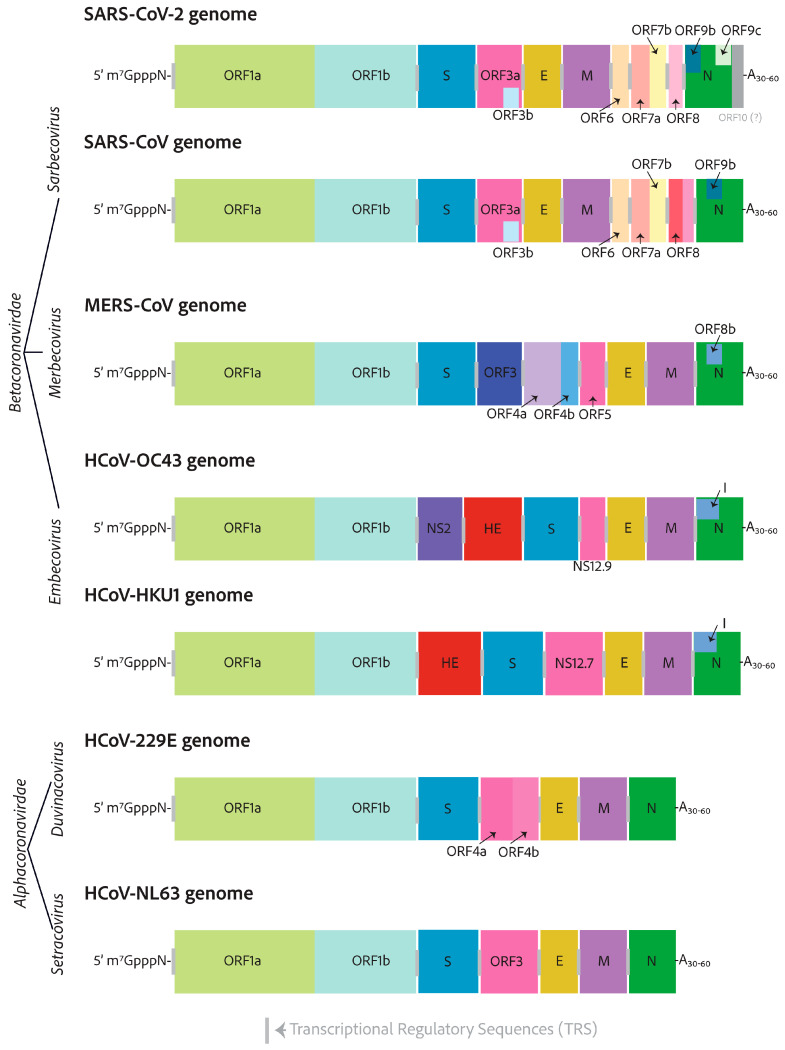
The RNA genomes of the seven human-infecting coronaviruses. The seven human-infecting coronaviruses are the alphacoronaviruses, HCoV-229E and HCoV-NL63, and the betacoronaviruses, SARS-CoV-2, SARS-CoV, MERS-CoV, HCoV-OC43, and HCoV-HKU1. All coronavirus RNA genomes have a 7-methylated-guanosine cap linked via a 5′–5′ triphosphate bridge mimicking, that of the host cell mRNAs. Additionally, the genomes have 3′ poly-A tails for mRNA protection from host cell immune defenses that de-cap un-polyadenylated mRNAs, leading to their eventual degradation. Alphacoronavirus lengths are approximately 27 kilobases, whereas betacoronaviruses are closer to 30 kilobases and have more open reading frames decorating their 3′-ends.

**Figure 3:**
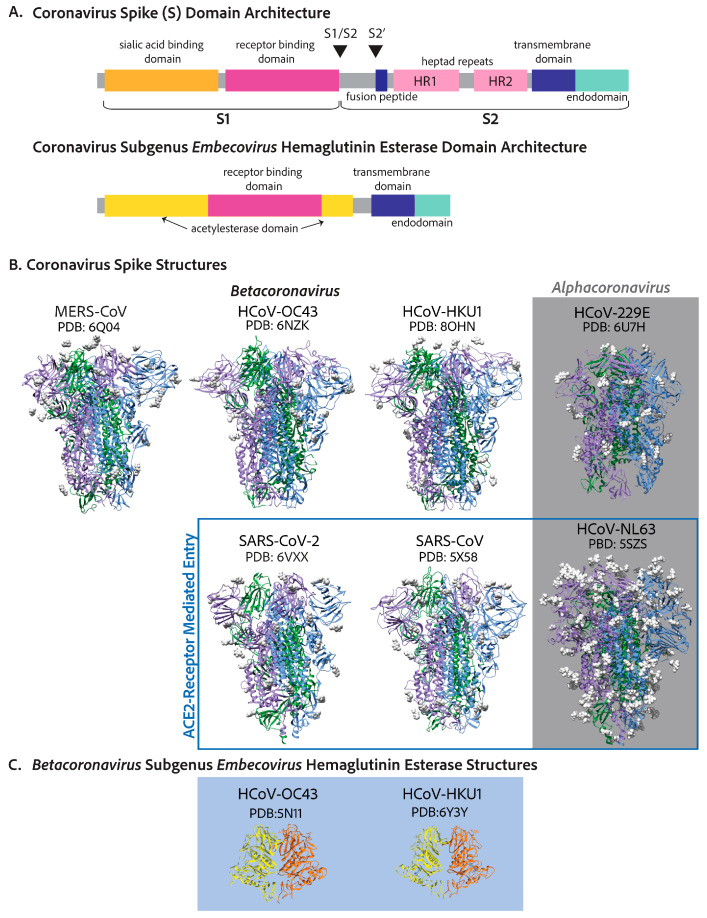
Coronavirus spike proteins have similar conserved domain architectures. (**A**) The betacoronavirus spike is designed to be cleaved at two sites, leading to two functionally different subunits: S1, which includes the receptor binding domain, and S2, the cell–virion fusion domain. The N-terminal domain of S1 is shown to interact with carbohydrates like sialic acids, and the receptor binding domain is in the C-terminal half of S1 [31,64,67,68]. S2 includes the fusion peptide, which buries into the host cell membrane, leading to cell–virion fusion, two heptad repeats, a transmembrane region, and an endodomain [30,38]. The endodomain includes ER-export (COPII), ER-retrieval peptides (COPI), and a membrane protein interaction site. (**B**) The spike crystal structures or cryo-EM structures of each human coronavirus. SARS-CoV-2, SARS-CoV, and HCoV-NL63 spike proteins all attach to ACE2 [69]. MERS-CoV interacts with DPP4 [67]. HCoV-229E interacts with APN [70]. Historically, HCoV-HKU1 was shown to use sialic acids as a functional receptor to trigger conformational opening to allow for S2’ cleavage and fusion [71]; however, it was recently shown that the HKU1 spike uses TMPRSS2 as a functional receptor with important sialic acid attachments [72]. HCoV-OC43 has only been shown to attach to sialic acids [73]. (**C**) The hemagglutinin esterase structures of the embecoviruses: HCoV-OC43 [74] and HCoV-HKU1 [75].

While the genomes of the seven human coronaviruses are evolutionarily distinct and differ mostly in the 3′ region (Figure 2), the entry mechanisms and what follows once inside the cell are similar. The coronavirus spike protein first attaches to a high-affinity binding partner found on the surface of a susceptible host cell [30]. This high-affinity binding partner is dubbed the cellular adhesion or attachment receptor/factor. The attachment of the virion to the cell surface can induce one of two mechanisms of cellular entry, both involving spike cleavage [26]. (1) If spike proteins on the virion are in close contact with cell surface cleavage proteins, the spike is first cleaved at site S1/S2 by a surface protease, as happens with furin for SARS-CoV [35]. For some coronaviruses, this step can happen in the virion-producing cell and for others it occurs at the cell surface of the target cell [30]. In both cases, the spike is cleaved at site S2’ by a cell surface protease, as happens TMPRSS2 for SARS-CoV-2 (Figure 3A) [30]. This cleavage at both sites, S1/S2 and S2’, activates the fusion of the cell and the virion membranes. Notably, spike proteins of alphacoronaviruses, HCoV-NL63 and HCoV-229E, only have one cleavage site at the S2’ location, and the two subunits S1 and S2 remain bound throughout cell entry [26,35]. (2) However, it is also possible that once the virion attaches to the cell adhesion factor, endocytosis will be induced [30,36,37]. After endocytosis, the cleavage of the spike will occur due to an endocytic protease, like cathepsin B or L, inducing the membrane fusion of the endosome and the virion [30,36,37]. For all coronavirus spikes, S2’ cleavage exposes the hydrophobic fusion peptide, which is inserted into the host cell membrane while the transmembrane domains of the three protomers are squarely attached to the virion membrane [38]. A hairpin-like formation pulls the two membranes together, leading to lipid mixing and hemifusion [38]. Finally, a fusion pore between the virion and the cell opens, releasing the contents of the virion into the host cell cytoplasm [38].

The virions encapsulate the viral genome, which is +ssRNA covered in nucleocapsid. There is evidence that the nucleocapsid plays a role in viral genome replication and transcription, likely acting as an RNA chaperone [39,40,41,42]. No viral replication proteins are housed inside the virion. Instead, the host cell cytosolic ribosomes translate them directly from the viral +ssRNA [43,44]. Two 5′ end open reading frames, ORF1a and ORF1b, are separated by a distinct secondary structure, causing a −1 ribosome frameshift in which translation produces either polypeptide 1a (pp1a) or the full polypeptide 1ab (pp1ab) [45,46]. These polypeptides are then self-cleaved by two proteases, the Papain-like 3CL-protease domain of nsp3 and the main protease (nsp5), resulting in 16 nonstructural proteins (nsp1–16) [47]. These proteins have roles in replication, transcription, immune evasion, and membrane reorganization. Many nsps form the replication and transcription complex (RTC) responsible for replicating the genome [48]. Nsp3, 4, and 6 are involved in forming double-membrane vesicle (DMV) structures that bud off cellular membranes, as occurs from the endoplasmic reticulum [49]. DMVs serve as a shelter that allows the RTC to replicate and transcribe the genome, resulting in double-stranded RNA intermediates that are protected from host cell immune responses by the DMVs [50]. The DMVs are also thought to concentrate the necessary components and host factors for the replication and transcription of the genome [51]. How the RTC, host factors, and +ssRNA enter these DMVs is unknown. However, there are pore structures formed by nsp3 that could allow materials to enter and exit [52]. The +ssRNA is first replicated into the -ssRNA, which is used as a template to synthesize new viral genomes [53,54]. Additionally, 3′ end structural and accessory proteins are transcribed in a discontinuous method of synthesis in which transcriptional regulatory sequences match with the 5′ end of the genome leader sequence, resulting in template switching [53,54]. The result is stepwise subgenomic mRNAs (sgmRNA) that each receive the 5′ end of the genome, a 5′ cap, all of the 3′ end genes after the template matching site, and the poly-A tail [53,54]. The +ssRNA genome and +sgmRNAs are released from the DMVs into the cellular cytosol [53,54]. This transcription method could allow for copy number control of the 3′-end structural and accessory proteins since only the first open reading frame of the sgmRNA is translated [53,54].

The spike, envelope, and membrane proteins are translated into the rough ER and follow into the ER–Golgi intermediate compartment, where both E and M are thought to induce the membrane curvature necessary for virion formation [28]. As the virion buds, E and M interact with and allow the membrane engulfment of the nucleocapsid-coated genomic RNA, forming a complete infectious virion [55]. While it was previously thought that the new virion egresses through the secretory pathway in a smooth-walled vesicle, recent evidence has shown that virions egress through deacidified lysosomes in a non-lytic manner [56].

## 3. Genomes and Basic Genetic Differences

Human coronaviruses are relatively distantly related and are closest in terms of relation to zoonotic ancestors [57,58]. With time, there have been evolutionary adaptations enabling them to infect new host species. The remaining similarities between human coronaviruses can teach us about the components that are most important to getting the job done [59]. All coronavirus genomes are 5′-capped and have a poly-A tail on their 3′ end [26]. They have very similar 5′-ends, with two open reading frames separated by a ribosome frameshift secondary structure. The open reading frames on either side are called ORF1a and ORF1b [26]. Coronavirus 3′-ends are radically different in comparison, but they all encode the essential structural components of the virion particles—the S, E, M, and N proteins—and several diverging accessory factors, as shown in Figure 2 [60].

The accessory factors are the least well-studied coronavirus proteins. The sarbecovirus 3′ end regions are the most complex, with 10 to 13 potential coding sequences. On the other end of the spectrum, the HCoV-229E and HCoV-NL63 alphacoronaviruses only encode the necessary structural genes—S, E, M, and N—as well as a viroporin-encoding gene, indexed as ORF3 for HCoV-229E, which is broken into two open reading frames for HCoV-NL63, ORF4a, and ORF4b [61]. All HCoVs encode a similar viroporin protein, whose gene is positioned between the S and E in the genomes, with many differing pseudonyms, all shown in pink in Figure 2 [61].

The HCoV-OC43 NS12.9 viroporin was knocked out in a recombinant mutant virus and found not to be essential, but it reduced viral titer 10-fold. It was thus suggested to be important for proper viral fitness and production [61]. NS12.9 was shown to have ion channel activity. This was confirmed with electrophysiology experiments with exogenous expression in yeast and Xenopus oocytes [61]. Viroporins from other enterovirus and influenza A, EV71-2B, and IAV-M2, respectively, could reverse the phenotype observed for the loss of NS12.9 in HCoV-OC43 infection [61]. Additionally, the SARS-CoV-2 ORF3a, HCoV-229E ORF4a, and HCoV-NL63 ORF3 were also able to complement the loss of NS12.9 in HCoV-OC43 infection [61]. Other similar accessory factors between distantly related betacoronaviruses include the I protein, which is situated in the middle of the nucleocapsid open reading frame. For SARS-CoV-2, the I protein is annotated as ORF9b, which has been shown to target the mitochondrial outer membrane protein Tom70 to switch off the mitochondrially regulated apoptosis responses and to target mitochondrial innate immune responses led by the release of mitochondrial DNA or the activation of the mitochondrial antiviral signaling protein, MAV [62]. MERS-CoV ORF8b was also shown to inhibit the innate immune response type I interferon expression by inhibiting the interaction of the IKKε kinase with HSP70, which is essential for the kinase activation of the interferon regulatory factor 3 [63].

## 4. Cell Entry

The coronavirus spike protein (S) forms trimer conformations that jet out from the virion membrane by approximately 20 nm [26]. While the initial binding to the corresponding adhesion factor determines the specificity of a cell in terms of being susceptible to infection, this is only the beginning of its function as a viral fusion protein [30,31]. After attachment to a susceptible cell, the spike is cleaved in highly conserved sites despite the receptor binding sequences of the protein being radically different in terms of amino acid sequences [31]. The cleavage of the coronavirus S protein leads to a conformational change akin to a loaded spring that launches membrane fusion between the CoV envelope and the cell plasma membrane [30]. The spike can be divided into two main domains—the S1 domain, responsible for engaging with host cell receptors, and the S2 domain, which fuses membranes—as shown in Figure 3A [30]. Cleavage leading to the separation of S1 and S2 can occur in either the virus-producing cell directly after translation or once they reach the cell surface of the next target [30]. SARS-CoV-2 and MERS-CoV S proteins are cleaved at the S1/S2 site in the virus-producing cell by a proprotein convertase like furin so that S1 and S2 domains of the spike of a mature SARS-CoV-2 or MERS-CoV virion are noncovalently bound subunits [30,35]. S1 is responsible for engaging the adhesion factor, and S2 anchors the virion to the membrane and facilitates membrane fusion [30,31]. S1 can engage carbohydrates such as sialic acids in its N-terminal domain and the protein receptor in its C-terminal domain [31]. The role of both carbohydrate and protein receptors is still being elucidated. However, there is evidence that both are important [64]. S is cleaved a second time at the S2’ location which is mediated by TMPRSS2 at the cell surface or by a similar protease on the endosome membrane like cathepsin L [30,31]. This second cleavage launches the spring, leading to membrane fusion and the release of the genome into the cytosol [38]. In contrast to SARS-CoV-2, SARS-CoV S protein is cleaved at both sites by the target cell proteases [65,66]. In either case, membrane fusion depends on the target cell proteases [30]. Figure 3B shows the solved crystal or cryo-EM structures of each human-infecting coronavirus spike protein, each decorated with glycosides, as well as the solved structures of the two human-infecting embecovirus hemagglutinin esterase proteins.

Adhesion factors for human coronaviruses follow some interesting trends. The angiotensin-converting enzyme 2 is the adhesion factor of SARS-CoV, SARS-CoV-2, and, through completely differential evolutionary means, HCoV-NL63 [36,76,77,78,79]. Thus, HCoV-NL63 is an important, safer alternative to SARS-CoV and SARS-CoV-2 in cell entry mechanism studies and the discovery of cell entry inhibition antivirals [78]. The functional receptor for MERS-CoV is the surface metallopeptidase, dipeptidyl peptidase 4 (DPP4) [80]. HCoV-229E utilizes aminopeptidase N (APN) as its functional receptor [70]. ACE2, DPP4, and APN are all members of the MA metallopeptidase family and have some similarity in tissue expression and structure (Table 1). More evidence is needed to ascertain the advantages of utilizing metallopeptidase for cell adhesion, as typically the spike is not cleaved via the attachment receptor [72,80]. HCoV-OC43 and HCoV-HKU1 spike proteins recognize the attachment of O-acetylated sialic acids to cell surface glycoproteins and glycolipids, which have been shown to allow cell entry. Numerous reports show that SARS-CoV, SARS-CoV-2, and MERS-CoV S proteins interact with sialic acids, although typically, the focus is the protein receptor that determines cell susceptibility [64,67,68,73,81,82].

While it has long been accepted that embecoviruses HCoV-OC43 and HCoV-229E attach to and recognize 9-O-acetylated sialic acids in receptor-mediated entry [73], it is not clear whether there are specific sialic-decorated proteins or lipids necessary for entry [72]. Saunders et al. recently showed that TMPRSS2 is a functional adhesion receptor for HCoV-HKU1 [72]. Using current methods, HCoV-HKU1 does not culture well in isolated cell culture models, making infection studies more difficult and HCoV-HKU1 the least studied coronavirus [72]. Because of this difficulty, Saunders et al. exogenously expressed HCoV-HKU1 spike in HEK293T cells that do not express TMPRSS2 [72]. Only when there is co-expression of TMPRSS2 is there cell–cell fusion. For the SARS-CoV-2 spike expressed in HEK293T cells, only the expression of ACE2 leads to cell–cell fusion and not TMPRSS2 expression [72]. They tested a full panel of other cell membrane proteases, including APN, DPP2, ACE2, TMPRSS4, and TMPRSS11D, but only TMPRSS2 allows cell–cell fusion for HCoV-HKU1 spike [72]. They also expressed TMPRSS2 protease-inactive mutants as well as wild-type in HEK293T cells and determined the entry of a lentiviral pseudovirus decorated with the HCoV-HKU1 spike, all of which still led to the cell entry of the pseudovirus likely via endocytosis means [72]. However, HEK293T without TMPRSS2 did not allow the entry of the HCoV-HKU1 pseudovirus, suggesting that TMPRSS2 is acting as a cell adhesion factor, not only as a spike cleavage protease, in the case of HCoV-HKU1 [72]. Intriguingly, Vero-E6 cells that express TMPRSS2 are not susceptible to infection with HCoV-HKU1 pseudovirus, indicating other cell entry factors such as protein sialyation or glycosylation must be necessary for proper HCoV-HKU1 entry [72]. To test this, Saunders et al. treated V20S cells that express TMPRSS2 with Neuraminidase to enzymatically remove protein sialic acids, specifically Lectin SNA and Siglec-E, without interfering with the cell surface levels of TMPRSS2 and found that, indeed, sialic acids were necessary to trigger HCoV-HKU1 entry [72].

The conclusions of this paper only focus on HCoV-HKU1, the least studied coronavirus. However, the implication that TMPRSS2 can be a cell adhesion factor outside its role in spike cleavage should be investigated for other coronaviruses, specifically HCoV-OC43, the coronavirus most closely related to HCoV-HKU1. There may be evidence that factors other than sialic acids are important for HCoV-OC43 receptor-mediated entry. The sialic acid binding domain of the HCoV-OC43 spike protein is in the S1 N-terminal domain; however, Chuyun Wang et al. found two S1 C-terminal domain-targeting neutralizing antibodies that inhibit infection but not sialic acid binding [83]. Nevertheless, the finding by Saunders et al., that only TMPRSS2 decorated with the correct sialic acids can allow the entry of the HCoV-HKU1 pseudovirus, highlights the potentially more important role of sialic acids in coronavirus entry, which should be investigated [72]. While protein receptors like ACE2 and adhesion factors like sialic acids receive a lot of attention, the cell proteases that induce spike cleavage can be equally important in terms of cell entry [84].

**Table 1:** Human coronaviruses and their primary cell tropism within the human body.

Virus	Host of Origin ^a^	Intermediary Host ^b^	Primary Cell Attachment Receptor/Factors ^c^	Human Cell Tropism/Attachment Receptor Tissue Expression ^d^	Susceptible Cell Lines ^e^
SARS-CoV-2	Bat	Pangolin (?)	Angiotensin-converting enzyme (ACE2)	Correlates well with ACE2 expression: ciliated epithelial cells as well as vascular endothelial cells of the lung, trachea, intestine, skin and sweat glands, kidney, brain and heart.	Vero E6/76, Calcu-3, Caco-2
SARS-CoV	Bat	Palm Civet	Angiotensin-converting enzyme (ACE2)	Correlates well with ACE2 expression: ciliated epithelial cells as well as vascular endothelial cells of the lung, trachea, intestine, skin and sweat glands, kidney, brain and heart.	Vero E6, Calu-3 cells
MERS-CoV	Bat	Camel	Dipeptidyl peptidase 4 (DDP4 or CD26)	Correlates well with DPP4 expression: expression in parathyroid gland, intestine, placenta, prostate, seminal vesicle, salivary gland and liver. Only low and medium expression in the nasopharynx and bronchus, respectively.	Huh-7 cells, Vero E6
HCoV-OC43	Mice	Bovine	9-O-acetylated sialic acids	9-O-Ac were found in the adrenal gland, colon, cerebellum, gray matter, pancreas, prostate, salivary gland, and small intestine, which correlates well with expression of CASD1 and SIAE, the O-acetyltransferase and O-acetylesterase, respectively. Both of these have low and medium expression in the nasopharynx and bronchus, respectively.	Vero E6, MRC-5, Mv1Lu, RD, Huh7, modified Vero E6 that stably overexpress TMPRSS2
HCoV-HKU1	Rodent	Mice	TMPRSS2, 9-O-acetylated sialic acids	TMPRSS2 localizes to the lung, gastrointestinal tract, pancreas, kidney, parathyroid gland. 9-O-Ac were found in the adrenal gland, colon, cerebellum, gray matter, pancreas, prostate, salivary gland, and small intestine, which correlates well with expression of CASD1 and SIAE, the O-acetyltransferase and O-acetylesterase, respectively. Both of these have low and medium expression in the nasopharynx and bronchus, respectively.	Primary Lung Epithelial Cultures, does not take well to isolated cell lines.
HCoV-NL63	Bat	Palm Civet	Angiotensin-converting enzyme (ACE2)	Correlates well with ACE2 expression: ciliated epithelial cells as well as vascular endothelial cells of the lung, trachea, intestine, skin and sweat glands, kidney, brain and heart.	Vero E6, MRC-5, LLC-MK2, Mv1Lu
HCoV-229E	Bat	Bat	Aminopeptidase N (APN)	APN is expressed on the surface of myeloid progenitors, monocytes, granulocytes, myeloid leukemia cells, and stem cells. It is highly expressed in the gastrointestinal tract, the liver, pancreas, and kidney.	MRC-5, Huh7, Mv1Lu

^a^ The host species of origin of a coronavirus is the species that has an endemic infection with the most closely related coronavirus ancestors [57,58,85,86]. ^b^ Coronaviruses have mosaic genomes with spliced regions from different coronaviruses, some of which may be of differing host species. If this splicing is in the spike region, the coronavirus may be capable of expanding its host species range, leading to cross-species transmission to the human host [57,58]. There are controversial opinions on whether SARS-CoV-2 was spliced in the spike region and even whether it has pangolin coronavirus ancestors as indicated by a “?” [1,87]. The intermediary host showed evidence of coronavirus infection with closely related ancestors of the human coronavirus and may have allowed for genomic recombination or adaptations leading to the capability of spreading to the human host. ^c^ The primary cell attachment receptors for SARS-CoV-2 [76,88], SARS-CoV [79], MERS-CoV [80], HCoV-OC43 [73], HCoV-HKU1 [71,72], HCoV-NL63 [78], and HCoV-229E [70]. ^d^ The human cell tropism is largely determined by tissue expression of the protein receptors—ACE2 [89,90], DDP4 [89,90], 9-O-acetylated sialic acids [89,90,91,92,93], TMPRSS2 [89,90], and APN [89,90]. ^e^ The most appropriate cell lines for culturing are those that are both susceptible to infection and produce an observable cytopathic effect that can be utilized to quantify viral titers. The more pathogenic coronaviruses have been known to produce drastic cytopathic effects in various cell lines—SARS-CoV-2, SARS-CoV [79,94], and MERS-CoV [51,95]. For endemic coronaviruses that were initially cultured in organ cultures of many different cell types, finding an isolated cell culture that achieves an observable cytopathic effect has been a challenge [14,23,24,96]. An isolated cell culture model for HCoV-HKU1 has still not been found. As such, it is typically cultured in primary epithelial cultures [72].

## 5. Evolution and Host Tropism—Cell Receptors and Cell Tropism

Coronaviruses are zoonotic, and many are capable of infecting multiple host species. The most closely related ancestors to human coronaviruses are those that infect other mammalian host species [57,58]. Host tropism, or the span of host species susceptible to infection, is primarily decided by the tissue expression of the primary adhesion factor recognized by the spike’s receptor binding domain as shown in Table 1. Recent evidence suggests that cell surface proteases, endosomal proteases, and the S2 domain are also important determinants of host tropism [31]. Cross-species transmissions of coronaviruses occur frequently, as we have seen in the past 20 years with SARS-CoV, MERS-CoV, and SARS-CoV-2 and will likely continue to see with human globalization and climate change impacts. What allows coronaviruses to expand their host range quickly? Genomic recombination may be one of the key drivers of host switching by coronaviruses, primarily when it occurs in the spike gene region [97]. The emergence of SARS-CoV as a human pathogen was likely a result of a recombination event, in which ancestral bat SARS-CoV-like viruses did not have the ability to recognize ACE2 and likely gained that ability through recombination on multiple occasions [97]. The bat pathogen able to infect humans had evidence of recombination with a palm civet coronavirus, making the palm civet the intermediary host species [57]. It has been shown that SARS-CoV-like bat viruses that do not have the ability to bind ACE2 are unable to infect the human host [98].

However, whether genomic recombination is necessary for a host-switching event is debatable. While all seven human coronaviruses have traces of genomic recombination shown by their mosaic-like genomes—the regions of different ancestral coronavirus sources stitched together—there is disagreement as to whether all human coronaviruses show evidence of that stitching in their spike gene, specifically for SARS-CoV-2 [97]. SARS-CoV-2’s most closely related ancestor is a bat coronavirus RaTG13, and some have suggested there is evidence of recombination with a pangolin coronavirus in the receptor binding domain of the spike [1,87]. Others argue that recombination was not a driving force in the spike region [99]. While pangolins may have acted as an intermediary host, some make the case that the most recent common ancestor of RaTG13 and SARS-CoV-2 evolved from closely related Sarbecoviruses in bats that had the ability to replicate in both pangolins and humans [87,99,100]. In this case, there was no recombination with a pangolin virus whatsoever. Indeed, the RaTG13 spike can also bind the ACE2 receptor, which it primarily utilizes in cell entry [99]. Thus, it may be the case that recombination was not a driving force in SARS-CoV-2 cross-species transmission, leading to the debate around whether the pangolin is an intermediary host.

Other hypotheses for how coronaviruses expand their host range include promiscuous receptor binding as well as infection and adaptation to intermediary hosts [97]. With the increased research efforts to understand and prevent SARS-CoV-2 infection, many noncanonical receptors have been identified for SARS-CoV-2, suggesting a potentially more receptor-independent cell entry mechanism. CD147 [101], Neuropilin-1 [102], AXL [103], GRP78 [104], TfR [105], and TMEM106B [106] have all been identified as noncanonical functional receptors for SARS-CoV-2 entry. While this receptor promiscuous cell entry has yet to be established as a means of host jumping, it is associated with the broad tissue tropism that presents with SARS-CoV-2 infection, which can often even be found in tissues with very low to no expression of ACE2 [107]. Broader tissue tropism has yet to be explored for endemic human coronaviruses.

Additionally, coronaviruses can quickly evolve and adapt to a new host, as we have seen with SARS-CoV-2’s rapid adaptation to the human host through beneficial mutations that arise randomly and are selected for by enhanced transmission and increased viral progeny, leading to variants that outcompete all others. This selection of beneficial mutations, leading to the rise of variants, is not only a trait of SARS-CoV-2. The molecular epidemiology of HCoV-OC43 suggests that different genotypes or variants evolved over time [108], albeit much slower than occurred with SARS-CoV-2. However, the initial stages of HCoV-OC43 infecting the human host may have resulted in a similar pace of mutational adaptation compared to SARS-CoV-2. Unfortunately, the molecular epidemiology of HCoV-OC43 may be lost to time. Historical records show there was a pandemic around the same time in which HCoV-OC43 first infected humans, as determined by molecular dating methods [57,58,109].

## 6. Pathogenicity

### 6.1. How Prevalent Are Endemic Coronaviruses?

Measuring the prevalence of endemic coronaviruses prior to the COVID-19 pandemic was complicated as mere common cold symptoms typically do not elicit a need to seek medical assistance, and thus many infections went undetected, leading to wide-ranging estimates of total endemic coronavirus prevalence from 5% to greater than 30%. Additionally, inherent seasonality and changes year to year in infection rates may have led to discrepancies between studies. Endemic coronaviruses have been studied in large cohort studies, such as in the Michigan Household Influenza Vaccine Evaluation (HIVE) study, which followed families with children who reported acute respiratory illness within the household from years 2010 to 2018 [110]. From 2010 to 2018, the HIVE cohort ranged from 895 to 1426 individuals. Of all acute respiratory illnesses reported, human endemic coronavirus tests were positive between 8.3% and 14.8% of the time, depending on the year, with HCoV-OC43 being the most prevalent overall, although in some years other coronaviruses were dominant [110]. Endemic coronavirus infections follow seasonality and peak every 2 years [110]. One explanation for this is that antibodies from the previous season can prevent symptoms of infection the following year [110].

The HIVE study followed already enrolled participants during the beginning of the COVID-19 pandemic, in which 437 patients reported a total of 773 acute respiratory illnesses between March 2020 and June 2021 [111]. The incidence of acute respiratory illness during this period was 50% lower than in the previous pre-pandemic length of time from March 2016 to June 2017 [111]. In this pandemic-era study, rhinovirus infection was the most prevalent, followed by endemic coronaviruses, and HCoV-NL63 was the predominant coronaviral infection, seen in 4.2% of all samples tested [111]. There was even one case of co-infection with NL63 and SARS-CoV-2 [111]. The start of this study coincided with the early waves of the pandemic, with the implementation of public health restrictions such as school closure, masking requirements, and the cancellation of large crowd events like concerts. Results suggested that these measures also abated endemic coronavirus infection [112].

The National Respiratory and Enteric Virus Surveillance System (NREVSS) followed the seasonality of endemic HCoVs from 2014 to 2021 [113,114]. The surveillance system collected viral testing results across the United States weekly from patient centers, including clinical, public health, and commercial laboratories [113,114]. Of all specimens submitted to the NREVSS, 3.6% were positive for any HCoV, and HCoV-OC43 was dominant, with 40.1% of all positive for an HCoV [113,114]. Shah et al. also reported on seasonality, suggesting that endemic HCoVs had seasonal onsets between October and November, peaking from January to February, with offsets in April to June [113]. The only year to deviate was 2020–2021, in which the endemic HCoV season was delayed by 11 weeks, likely due to public health measures taken to reduce the SARS-CoV-2 burden [113]. While this study is valuable for detecting seasonal onset and offset of endemic coronaviruses, it may be biased because only those who deem themselves ’sick enough’ will seek medical assistance and go through viral testing [113,114].

Wastewater studies may be a better approach with which to measure endemic coronavirus prevalence as there is no bias in terms of the severity of illness needed to report, and they would also account for asymptomatic cases where the virus may still be shed [115]. However, fewer data are attached, and no information is given about who is more likely to be infected or have more pathogenic disease, such as what age groups are affected [115,116]. Other information can supplement wastewater studies about the region, such as high tourism levels and population demographics, which should be considered when interpreting results [115,116]. Additionally, more studies are needed to test how long viruses ‘survive’ in wastewater to then be detected some number of days later [115,116]. Wastewater testing was a valuable resource in seeking to determine when SARS-CoV-2 was peaking in the past years of the COVID-19 pandemic; as a result, other respiratory viruses are now being surveilled with this method [115,116]. Bohem et al. tested wastewater RNA concentrations via RT-qPCR analysis of several respiratory viruses, including human influenza, metapneumovirus, parainfluenza, endemic coronavirus, respiratory syncytial virus, rhinovirus, and SARS-CoV-2, from a Californian wastewater treatment plant from February 2021 to June 2022 [116]. During this time, SARS-CoV-2 had the highest median viral RNA concentration found in wastewater [116]. Endemic coronaviruses had the second-highest median viral RNA concentration, above that of rhinovirus [116]. This wastewater study contrasts with the self-reported HIVE and NREVSS studies that show that rhinovirus is the most abundant [112,113,116]. Gathering information about endemic coronaviruses by both means is vital to understanding endemic coronavirus prevalence better.

### 6.2. What Kind of Disease Do Endemic Coronaviruses Cause?

The first observation of human coronavirus disease was in the 1960s, when healthy adults volunteered to be infected with HCoV-229E and HCoV-OC43 [3,117,118]. Both were shown to cause a common cold that, on average, cleared within one week [3,43]. Symptoms from infected adults showed that the alphacoronavirus, HCoV-229E, was typically mild compared to the betacoronavirus, HCoV-OC43 [3]. Nevertheless, these studies left out vulnerable populations, including children. More recent studies show that endemic coronavirus infection is more prevalent in children under the age of 5, with roughly 42% to 50% of 6–12-month-olds displaying antibodies from an HCoV-229E infection [119], and that number increases to 65% for 2.5–3.5-year-olds [120]. Other studies show a similar trend where endemic coronaviruses tend to be more severe in children than adults, likely because adults have built up neutralizing antibodies and T-cell responses [121,122]. HCoV-NL63 was first discovered in an infant with pneumonia due to endemic coronavirus infection [10,11,69]. Other possible complications in infants or children due to endemic coronavirus infection are croup, encephalitis, and bronchitis [3]. In addition to children, patients who are immunocompromised or are elderly can have more severe outcomes to endemic coronavirus infection [3,9,123]. HCoV-HKU1 was discovered in 2004 in an elderly Chinese man who presented with chronic obstructive airway disease [12]. In general, infection by an endemic coronavirus is not lethal or severe in adults without underlying disease or old age [3]. However, cases in infants, children, and those with underlying diseases can be more complicated.

Zoonotic coronaviruses are known to cause disease in the respiratory tract and central nervous system, as well as gastroenteritis [3]. While human coronaviruses can be detected in stool samples, as described in the wastewater studies in the earlier section, no HCoV-229E- or HCoV-OC43-infected volunteers presented gastrointestinal disease, suggesting that endemic coronaviruses may be shed through the gastrointestinal system without causing considerable disease [3,5]. Other studies reveal that there can be some gastrointestinal symptoms in cases of human endemic coronaviruses, specifically in children [124]. There has also been evidence of endemic HCoV infection of the central nervous system in mice models, and this has been found in human post-mortem brain samples [125,126,127]. Some argue that the effect of human endemic coronavirus on the central nervous system has been severely underestimated [128].

In contrast to endemic coronaviruses, SARS-CoV, the causative agent of severe acute respiratory syndrome, causes considerable pathology, even in healthy individuals [6,7,8,129]. The SARS-CoV epidemic, although brief, spread to 29 countries with a total of 8422 cases and 916 fatalities, roughly a 10% fatality rate [129]. The spread of SARS-CoV was controlled within 7 months of the first outbreak. SARS disease follows a two-phase, occasionally three-phase, illness in which the first phase is typically symptom-free, followed by the second phase of respiratory symptoms, fever, vomiting, and diarrhea [129]. Then, about 15% of adults will develop a third phase, in which there is acute respiratory distress [129]. Public health measures, including the quarantine of infected people and contact tracing, were integral in limiting the SARS-CoV epidemic [129].

The Middle East Respiratory Syndrome coronavirus, MERS-CoV, which initially became known in 2012, causes the most severe disease of any known coronavirus, with a mortality rate of 35% per the World Health Organization [130]. The transmission of MERS-CoV typically occurs with interaction with a dromedary camel; however, human-to-human transmission is possible and more likely to occur among those who provide health care to an infected individual [130]. Symptoms of MERS-CoV infection typically include a fever, cough, shortness of breath, and pneumonia [130]. Gastrointestinal symptoms have also been reported [130].

SARS-CoV-2 infection typically involves mild to moderate respiratory illness, and most people recover without specialized care [131]. However, some populations, like those with co-morbidities and seniors, are more likely to develop more severe disease and require medical intervention [131]. Symptoms usually begin 5–6 days after exposure and can last up to 14 days [131]. The most common symptoms are a fever, chills, and a sore throat. However, they may also include muscle aches, fatigue, a runny nose, headaches, dizziness, the loss of taste or smell, appetite loss, and gastrointestinal illnesses such as nausea, vomiting, and diarrhea [131]. Additionally, several patients who recover from COVID-19 disease develop chronic illness, which includes a wide range of symptoms that not present in the individual prior to SARS-CoV-2 infection, known as long COVID [131].

### 6.3. A Note on Endemicity

Endemic viruses are consistently maintained in a population and have predictable patterns [132]. For example, the flu peaks in winter, and the most prevalent strains are predicted from the previous year’s data to inform vaccine development [132]. Nonendemic viruses, on the other hand, have an unstable and unpredictable transmission with potential surges or disappearances over periods of time [132]. Despite its worldwide presence, SARS-CoV-2 has yet to reach an endemicity seen with the other HCoVs [132]. Endemicity is reached when there is a reduction in the overall virus transmission, and a pattern of infection emerges [132]. At this moment, SARS-CoV-2 population dynamics are unpredictable, unlike endemic viruses, and the threat of resurgence and variant transmission continues [132].

## 7. Replication and Transcription Proteins

**Figure 4:**
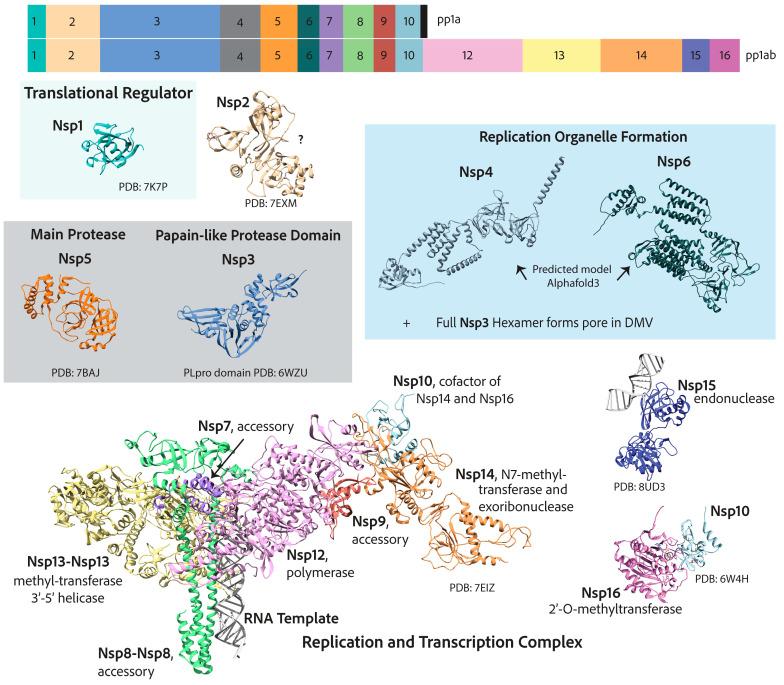
A map of coronavirus 5′-end nonstructural genes that, once translated into two polypeptides (pp1a and pp1ab), are self-cleaved into 16 nonstructural proteins that are responsible for genome replication and transcription. The function of nsp2 is not completely resolved. Nsp11, not shown, encodes a 13 amino acid peptide whose function is unknown.

**Figure 5:**
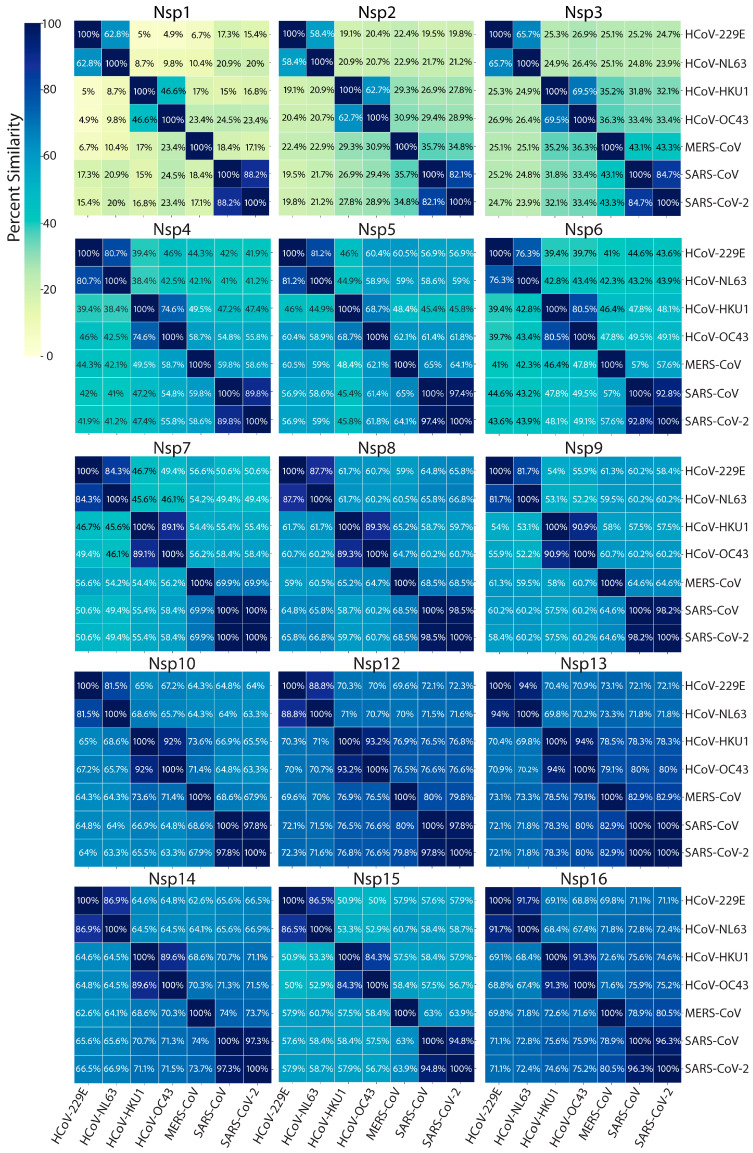
Heatmaps of percent similarity between human coronavirus nonstructural protein amino acid sequences. NCBI reference genomes used for sequence analysis: SARS-CoV-2 (NC_045512.2), SARS-CoV (NC_004718), MERS-CoV (NC_019843.3), HCoV-OC43 (NC_006213.1), HCoV-HKU1 (NC_006577), HCoV-229E (NC_002645.1), and HCoV-NL63 (NC_005831.2).

Arguably, all viruses have complex methods to deal with their genetic material, considering that they most likely require host factors to replicate while conflictingly needing to hide that genetic material from invader detection systems of the host cell. Coronaviruses are no different. After the translation of the two 5′ open reading frames and subsequent self-proteolytic cleavage by the main protease, nsp5, and the Papain-like 3CL protease domain of nsp3, the 16 nonstructural proteins get to work with three main tasks outlined below: (A) the reorganization of cellular membranes to form replication organelles; (B) viral genome replication and transcription inside these replication organelles; (C) RNA-capping [26,45]. A detailed map of the two coronavirus 5′-encoded polypeptides and the nonstructural proteins is depicted in Figure 4. Endemic coronaviruses are organized similarly to the more pathogenic coronaviruses and have considerably similar nonstructural proteins. The similarity of each nonstructural protein from the seven human coronaviruses is depicted with heatmaps in Figure 5. The most highly conserved nsps are those distal to the 5′ end, such as nsp10, nsp12, nsp13, nsp14, and nsp16. Accordingly, due to this high degree of conservation in amino acid sequence and function, endemic coronaviruses are favorable model systems for studying these nonstructural proteins’ functions and discovering inhibitors that can be used as therapeutics. Furthermore, nonstructural proteins involved in genome replication and transcription may be better targets for resistance-refractory therapeutics than highly mutagenic spikes.

### 7.1. Reorganization of Cellular Membranes to Form Replication Organelles

All mammalian-infecting positive-stranded RNA virus replication complexes can reorganize cellular membranes remarkably [133,134]. Different viruses tend to reorganize distinct specific organellar membranes to assemble their replication complexes. This has been shown for poliovirus [135,136], hepatitis c virus [137], norovirus [138], arteriviruses [139], and coronaviruses [51,52]. One of the earliest descriptions of coronavirus’ reorganization of organellar membranes to form replication organelles was of murine hepatitis virus (MHV), a mouse-infecting coronavirus [140,141]. These early papers hint at replication components being associated with double-membraned vesicles. These structures were characterized further in SARS-CoV-infected cells, showing that the replication complex components localize to DMVs that bud from the ER and Golgi membranes as seen by immunofluorescence and electron microscopy [142,143,144]. These ER–Golgi-associated vesicle structures where replication components localize are distinct from compartments that form new virion particles [51,143]. It was later shown that the transmembrane proteins nsp3, nsp4, and nsp6 could form these replication organelles when expressed in cells that were not infected [49]. These DMVs may protect viral genomic RNA during replication, which forms a double-stranded RNA intermediate that is easily recognized as foreign by the host cell Rig-I-like receptors [145]. Further, the compartmentalization of replication could enhance replication kinetics by increasing the concentration and availability of necessary viral and host factors [51].

Most recently, coronavirus DMVs were shown to not only house the replication complex but also to be the site of viral RNA synthesis by electron microscopy with radioactively labeled ribonucleotides for MERS-CoV, SARS-CoV, and infectious bronchitis virus (IBV) [51]. Interestingly, these replication organelles were shown to have pores formed by a hexamer of nsp3, which spans the double membrane [52]. Electron tomography of cryo-lamellae showed that the DMVs of MHV-infected cells have a membrane-spanning pore [52]. Using a GFP-tagged-nsp3 construct, the domain density in the electron tomography confirmed that nsp3 forms the pore complex [52]. Nsp3 has an N-terminal ubiquitin-like domain that binds single-stranded RNA (like the viral genome) [146] and the nucleocapsid [147]. There are still some great mysteries about these DMV replication organelles. How do nsp4, nsp6, and nsp3 form them? How does viral RNA enter? How do all the components of the replication complex enter, or is the complex formed inside?

### 7.2. Viral Genome Replication and Transcription Inside Double Membranous Organelles

The most complete structure of the coronavirus RTC consists of the RNA-dependent RNA polymerase (RdRp), the helicase, the bifunctional exoribonuclease and N7-methyltransferase protein, as well as several essential accessory factors [148]. The RNA-dependent RNA polymerase catalytic subunit, nsp12, has the typical right-handed conformation of polymerases [46,149]. Nsp12 only displays polymerase activity for short RNA templates with an RNA primer. Nsp12 interacts with nsp7 and a dimer of nsp8, which stimulate its polymerase activity [46,149]. It has also been suggested that nsp7 and nsp8 act together as a primase [150]. However, some suggest they are merely processivity factors [151]. Nsp7 and nsp8 have RNA-binding capability themselves [151]. The RdRp works closest with the helicase, nsp13, an ATP-dependent 3′ to 5′ helicase that has two copies in cryo-EM structures of the complex [152]. The 3′ to 5′ helicase activity opposes that of the RdRp, which adds nucleotides to the 3′ end [152], a contradiction that is yet to be fully parsed out [152]. Nsp13 is also involved in RNA capping by acting as an RNA 5′triphosphatase (RTPase), hydrolyzing the 5′γ-phosphate end of the nascent RNA [152]. Intriguingly, both nsp12 and nsp13 were characterized as FeS-cluster-containing enzymes and may be involved in electron relay cascades [153,154]. Two FeS clusters were found in nsp12 by Mössbauer spectroscopy, and the mutation of cysteine residues that ligate the cluster in the polymerase domain diminished polymerase activity [153,154]. Nsp13 uniquely ligates two zinc ions and one FeS cluster [154]. The replacement of the lone FeS cluster in nsp13 with zinc diminished the affinity of the helicase with its physiological substrate, dsRNA, instead increasing its affinity with dsDNA [154].

The most straightforward process the RTC undergoes inside the cell is continuous RNA synthesis. The viral positive RNA genome is first replicated to the negative sense, creating a double-stranded intermediate, the substrate of the helicase, nsp13 [54]. After unwinding, the negative sense strand is used as a template for synthesizing a new positive-sense viral RNA genome [53,54]. While simple, the act is incredible given the sheer size of the coronavirus genome, which can be three to four times the size of a typical RNA virus [149]. The speed with which the RdRp accomplishes this feat is also alarming. It is more than twice as fast as the poliovirus 3Dpol [149]. Generally, replication rates are inversely related to fidelity [155]. However, this is not the case for coronaviruses, whose RdRp has relatively comparable misincorporation rates to poliovirus 3Dpol, albeit slightly higher [149]. In vitro mutational studies attribute the high processivity rate with minimal loss of fidelity to an atypical alanine in the motif F active site of coronavirus nsp12 (as shown for SARS-CoV) and serine in motif C of the palm domain, respectively, as compared to the poliovirus 3Dpol [149].

Additionally, coronaviruses have a built-in proofreading exoribonuclease, nsp14, that cleaves misincorporated ribonucleotides. Nsp14 is in the DEDD family of 3′-5′ exonucleases and has been shown to be associated with the RdRp [148]. Nsp14 exoribonuclease activity is increased significantly in the presence of nsp10, a small cysteine-rich protein [148]. The substrate of the nsp14-nsp10 complex is dsRNA, which excises misincorporated ribonucleotides at the 3′ end [156]. Mutating the DEDD motif of MHV nsp14 of an infective virus results in an increased mutation rate of 15- to 20-fold higher than seen in a wild-type virus [157].

The second mode of function of the RTC is discontinuous RNA synthesis or the transcription of 3′-end structural and accessory factors [54]. This transcription relies on transcriptional regulatory sequences in the viral genomes between 3′ open reading frames that match a 5′ leader sequence [53,54]. During RNA synthesis, single-strand matching allows for template switching such that each newly synthesized 3′-end mRNA or subgenomic mRNA has both the 3′-end poly-A tail as well as the 5′-end up to the leader sequence, which is then capped, as discussed next [53,54].

### 7.3. RNA Capping

The coronavirus RNA-capping mechanism is a multi-enzyme process that mimics cellular RNA capping. It is essential for the virus to evade host cell immune 5′-3′ exonucleases that target uncapped RNAs, and it facilitates the translation of viral RNAs by cellular ribosomes [53]. After the synthesis of new viral genomic RNA or subgenomic mRNA, first, the 5′-most nucleotide γ-phosphate is hydrolyzed by nsp13 RTPase activity (pppA-RNA → ppA-RNA) [53]. Subsequently, the NiRAN domain of nsp12 acts as an RNA-guanylyl-transferase to transfer a guanosine monophosphate to the 5′-diphosphate RNA, resulting in a 5′–5′ triphosphate bridge or (GpppA-RNA) [53,158]. Nsp14, with its C-terminal N7-methyltransferase domain, is then recruited to methylate the N7 position of the guanosine, resulting in the cap-0 structure (7m-GpppA-RNA) [53]. Another methyl group is transferred to the 2’O position of the first mRNA nucleotide (7m-GpppAm2-RNA) by the S-adenosylmethionine (SAM) enzyme nsp16, with nsp10 as its cofactor [53]. This second methylation is important for preventing the reversal of the guanylyl transfer reaction [159].

### 7.4. Additional Functions of Nonstructural Proteins

Cellular translation repression by nsp1 was captured by cryo-electron microscopy to bind the 40S ribosomal subunit at the mRNA channel to inhibit translation in 2020 [160]. The repression of translation by nsp1 has two main advantages: (1) the inhibition of cellular innate immune responses and (2) the enhanced production of viral proteins [160]. There is evidence that viral proteins and critical host factors can bypass nsp1 translational repression, but how they do so is highly debated [161]. Bujanic et al. showed that specific residues within the first stem-loop of the leader sequences are both necessary and sufficient to bypass the repression of nsp1 translation [161]. Rao et al. determined that a 5′ terminal-oligopyrimidine is responsible for the evasion of viral translational repression [162]. Others have shown that multiple elements of viral RNAs enable this capability [160]. Nsp1 has also been shown to inhibit cellular innate immunity by catalyzing the cleavage of host cell defense mRNAs while protecting viral mRNAs by binding the 5′ leader sequence [163,164]. Ribosomal translation inhibition and the cleavage of host cell mRNAs by nsp1 are functionally distinct, as mutational studies of nsp1 are able to eliminate mRNA cleavage but not ribosomal inhibition [165]. Nsp15 is an RNA endonuclease that has also been linked to the degradation of cellular mRNAs and could be a target of therapeutics [166].

## 8. Recent Advancements in Endemic Coronavirus Culturing and Discoveries Found in Endemic Coronaviruses

Prior to the SARS-CoV epidemic in 2002, the lack of interest in endemic coronaviruses (at the time, only HCoV-OC43 and HCoV-229E) was due to their dismissal as common colds associated with children’s upper respiratory tract infections. SARS-CoV rallied scientific interest in human endemic coronaviruses, leading to the discovery of two more endemic coronaviruses, namely, HCoV-NL63 and HCoV-HKU1 [11,12,13]. However, there was another bottleneck in endemic HCoV research—a lack of reliable cell culture-based model systems and reproducible methods with which to study the viruses [14,167]. After the COVID-19 pandemic, there was a resurgence in interest in producing such model systems and methods to study the human endemic coronaviruses as a replacement for the more pathogenic coronaviruses [14,23,24,96].

### 8.1. Low Viral Yields Impeded Research Involving Endemic Coronaviruses

The first challenge human endemic coronaviruses pose is low viral yield from isolated cell culture models [14]. HCoV-OC43 was first cultured in an organ culture, as the name ‘OC’ suggests, that consisted of many different cell types. It was later passaged in HRT-18 or human ileocecal adenocarcinoma cells as the virus was isolated in a 67-year-old patient with adenocarcinoma [14,24]. While HCoV-OC43 can replicate in HRT-18 cells, the viral yields tend to be quite low [14,24]. When creating a viral stock, higher viral yields, in the range ≥10^6^, are essential for infection studies at a higher multiplicity of infection to create a more synchronized viral infection [14]. Many have approached this challenge by identifying different cell lines that may be more susceptible to infection by each human endemic coronavirus, although the resulting suggestions are quite heterogeneous [14,23,24,96,168].

### 8.2. Viral Quantification of Endemic Coronaviruses Proved Challenging

However, all odds are against the virologist. While the low viral yield is a key player in culturing these viruses, another issue is how one can accurately measure the infectious viral titer of a virus that does not produce a significant cytopathic effect (CPE) [167]. CPE is the detectable signs of cell death and changes in morphology related to viral infection. The first sign of CPE is the rounding of the cell body, but it can also include the development of inclusion bodies and even syncytia [169]. Virologists’ bread-and-butter techniques for quantifying viral titer are a plaque assay and the median tissue culture infection dose (TCID_50_) assay, both of which require considerable CPE [170]. The plaque assay includes infecting a lawn of cells in a well plate with a serially diluted virus [170]. After a predetermined number of days, a plug, typically made of agarose or another setting substance, is poured into the well so that when the plug is removed, any loose or dead cells are removed with it [170]. The number of spots or plaques where cells are removed is correlated directly with the number of infectious virus particles added to the well [170]. TCID_50_ is even more straightforward in theory. Typically, a 96-well plate of cells is infected with a virus stock that is serially diluted [171]. After a predetermined number of days in which CPE observations are at a peak, each well is binarily marked for CPE by microscope observation or staining of cell death [171]. The dilution at which 50% of the cell culture wells show CPE, known as the TCID_50_/mL, can then be calculated using the Reed–Muench equation, which is a standard method for determining infectious virus titer [172]. These assays assume that the number of infectious virus particles correlates with the CPE observed. This assumption has proved inaccurate in the case of endemic coronavirus infection in some cell types and conditions.

In 2008, an immunoperoxidase-based (IPA) assay was developed to label virus-infected cells with an antibody coupled with horseradish peroxidase (HRP) activity. The resulting HRP activity of the serially diluted virus was used to calculate the infectious virus titer [167,173]. This IPA assay determined HCoV-229E and HCoV-OC43 viral titers more accurately than others [167,173]. While this does result in a more accurate reading of virus titer for noncytopathic viruses, it is a tedious and often expensive process that never really caught on for use to assay virus titer [14].

### 8.3. Recent Advancements in Endemic Coronavirus Culturing and Quantification

With the history of endemic coronavirus culturing and the demand for a less pathogenic coronavirus infection model during the COVID-19 pandemic, scientists were motivated to optimize cell lines and techniques for endemic coronavirus infection, as itemized in Table 2. Fausto et al. optimized the HCoV-OC43 infection of Vero E6 cells, HCoV-229E infection of Huh-7 cells, and HCoV-NL63 infection of LLC-MK2 cells [14]. They additionally note that infection should take place at the more physiologically relevant 33 °C in the cooler areas of the upper respiratory and nasal areas, stating that HCoV-NL63 and HCoV-229E are severely attenuated at 37 °C [14]. For assays requiring a very high virus titer, they recommend the ultracentrifugation of the viral stock to increase the concentration [14]. Fausto et al. additionally outlines a detailed quantification method utilizing a standard plaque assay and a method to assess genomic/subgenomic RNA levels via RT-qPCR [14].

Schirtzinger et al. additionally show that HCoV-OC43 can induce CPE in Vero E6 cells at 3 days post-infection (dpi) [24]. Even though there is visible CPE, they note that there is no visible plaque in a plaque assay before 4 days post-infection and that there is some undefined plaque morphology in that the edges of plaques run into each other making exact quantification difficult [24]. The TCID_50_ assay is also performed and validated in this cell line. Schirtzinger et al. also identified MRC-5 cells as a candidate cell line for HCoV-OC43 infection, which was shown to induce CPE by 2 dpi [24]. HRT-18 cells did not have any CPE, even after 7 days of infection, and viral titers were determined by the immunoperoxidase assay validating the observations of decades of endemic HCoV research [24]. While MRC-5 cells may be better than HRT-18 cells, they can be slow-growing and challenging, as Bracci et al. discuss [168]. They instead suggested a mink lung cell line, Mv1Lu, which was shown to have CPE and viral titers of both HCoV-OC43 and HCoV-229E by an agarose-based plaque assay [168]. Hu et al. test several cell lines, including RD, MRC-5, BSC-1, Huh-7, and Vero E6, for HCoV-OC43, HCoV-229E, and HCoV-NL63 [96].

Another highly effective approach to endemic HCoV culturing was outlined by Hirose et al., who tested modified cell lines that stably express TMPRSS2, the protease responsible for S2’ cleavage and membrane fusion of many HCoVs [23]. This method is further supported by evidence that human coronaviruses may utilize TMPRSS2-mediated entry over the endosomal cathepsin protease entry mechanisms [23]. CPE-based assays of HCoV-OC43 infection of Vero-E6 cells with overexpressed TMPRSS2 showed viral titers most similar to those assessed by the non-CPE-based immunoperoxidase assay, while Vero-E6 cells without overexpressed TMPRSS2 exhibited reduced CPE, as previously reported [23]. Even with current technical advances, there is a lack of consensus on which cell line and quantification method is best. However, many recent studies show there have been surges forward in efforts to utilize endemic human coronaviruses to discover antivirals and understand coronavirus biology.

### 8.4. Examples of Endemic Coronaviruses as Surrogates for the More Pathogenic Coronaviruses in Antiviral Discovery

While Paxlovid, nirmatrelvir with ritonavir, is commercially available and efficacious as an antiviral treatment for SARS-CoV-2 infection, there is evermore a need to develop alternatives to keep up with peaks of infection during which population immunity may be low and opportunistic new variants that transmit quickly and efficiently [22]. Additionally, Paxlovid often has an unpleasant rebound effect in which individuals who, after taking Paxlovid, have an initial relief of symptoms only to regain those symptoms within a week later [22]. Furthermore, Paxlovid has numerous drug–drug interactions, with some of the most common medications making people who are more likely to have COVID-19 complications unable to receive the proper antiviral treatment [178]. While Paxlovid is efficacious, more alternative antivirals are still urgently needed. Endemic HCoV testing for antiviral therapeutics, as a surrogate for the more pathogenic coronaviruses, has prevailed as a promising initial screening method for drug compounds. This is specifically true for the use of HCoV-OC43, whose replication and transcriptional components are the most highly conserved with SARS-CoV-2. Several laboratories have adopted approaches akin to this method.

Ordonez et al. utilized an in vitro HCoV-OC43 infection model system in Vero C1008 cells to screen readily available compounds and determine unknown antiviral capabilities [179]. They identified Sulforaphane, an isothiocyanate, i.e., a plant-derived activator of the transcription factor nuclear factor erythroid 2-related factor (NRF2), describing its antioxidant and anti-inflammatory capabilities [179]. Sulforaphane inhibited HCoV-OC43 and SARS-CoV-2 at a median inhibitory concentration, IC_50_, of roughly 10 µM [179]. The cytotoxic concentration CC_50_ of Sulforaphane is around 80 µM. They additionally tested Sulforaphane in vivo against SARS-CoV-2 in K18 transgenic mice expressing a human ACE2 receptor (K18-hACE2) [179]. SARS-CoV-2-infected animals treated with Sulforaphane 30 mg/kg daily were better off in terms of bodyweight loss, the viral load in both the bronchoalveolar lavage and in the lung, and the histopathology severity score of the lung [179].

A similar approach was taken by Wang et al., who first utilized the molecular docking of FDA-approved drugs from the ZINC15 database, including a total of 1576 drugs, to the active site of the crystal structure of SARS-CoV-2 nsp5 or the main protease, Mpro, and an Itasser-modeled Mpro of HCoV-OC43 as a first-wave selection process [180]. Subsequently, compounds that were shown to bind Mpro in molecular docking experiments were tested in an infection model of HCoV-NL63, where researchers identified a novel pan-coronavirus antiviral, dyphylline, a xantheine derivative that works as a bronchodilator by relaxing smooth muscle tissue [180]. Dyphylline was shown to have an inhibitory effect in HCoV-229E, HCoV-OC43, HCoV-NL63, and SARS-CoV-2 infection, with an IC_50_ of around 50 µM [180]. While the prospective target of dyphylline is Mpro, researchers also suggest that there may be a synergistic effect in vivo, enabled by its function as a bronchodilator [180].

Erythromycin estolate (Ery-Est) is a macrolide antibiotic that has previously been shown to inhibit infection with several flaviviruses such as Zika virus, Dengue virus, and yellow fever [181,182]. Its efficacy against human coronaviruses was first established in an HCoV-OC43 infection model [181]. Ery-Est was shown to have an IC_50_ of 1.4 µM and a CC_50_ of >100 µM. Ery-Est was found to inhibit the virus particle directly [181]. The authors incubated the HCoV-OC43 virus directly with Ery-Est and then separated the virus from Ery-Est using the PEG-8000 separation technique. After separation, the virus was tested for infectivity in RD or HRT-18 cells in a plaque assay [181]. HCoV-OC43 treated with Ery-Est that was then separated from Ery-Est lost infectivity, while a negative control HCoV-OC43 treated with erythromycin (Ery) that was then separated had no effect on infectivity [181].

Feng et al. utilized another scheme designed to find inhibitors of the host cell essential translation factor eIF4F, in which they started with over 300,000 compounds that were selected by a high-throughput screening method in which the inhibition of both mechanisms of 5′cap-dependent and 5′cap-independent translation of mRNA in the treated cells is monitored by a luciferase reporter assay [183]. The authors point out that, like SARS-CoV-2 genomic and subgenomic mRNAs that utilize eIF4F as a translation factor, cellular mRNAs undoubtedly rely on this machinery for translation too [183]. However, during coronavirus infection, cellular mRNAs are translated at a much lower frequency, and prior research has shown that housekeeping proteins are much less affected by eIF4F inhibition than viral proteins or oncogenes [184]. The result of the first-wave screening was > 3000 compounds [183]. Of those, 221 compounds had 5′-cap-dependent translation inhibition and had low cytotoxicity in HCT116 cells [183]. Computational cheminformatics and commercial availability further narrowed the selection to 17 compounds [183]. These 17 compounds were assessed for antiviral activity during HCoV-OC43 infection of the A549 cell model [183]. Viral replication was determined by immunofluorescence and Western blotting for the nucleocapsid protein [183]. This study identified two eIF4F inhibitors that inhibit protein translation of HCoV-OC43 nucleocapsid at 10 µM [183]. This study highlighted the importance of a CPE-inducing quantification system as no IC_50_ was determined, likely due to the cost and duration of the immunofluorescence and Western blotting techniques.

While endemic coronaviruses are an excellent tool for safely and approachably investigating coronaviruses, even with the high degree of similarity between proteins, the effectiveness of an antiviral may not be transmutable between coronaviruses and, therefore, must always be tested. For instance, in 2014, Lin et al. discovered a novel inhibitor of the HCoV-OC43 nucleocapsid, PJ34, a poly ADP-ribose polymerase 1 (PARP1) inhibitor previously used against cancer [185]. They showed that PJ34 inhibited HCoV-OC43 and determined the crystal structure of the nucleocapsid N-terminal domain (N-NTD), establishing the mechanism of action of PJ34 as blocking the ribonucleotide binding site and thus reducing nucleocapsid RNA binding [185]. In the wake of the COVID-19 pandemic, PJ34 was tested for the inhibition of SARS-CoV-2 [186]. Unexpectedly, given the high degree of structural conservation between the two N-NTDs, PJ34 was shown not to be effective against SARS-CoV-2 infection with either the Wuhan or D614G variant [186]. Yamamoto et al. compared the HCoV-OC43 N-NTD and the SARS-CoV-2 N-NTD, each in complex with the ribonucleotide UMP [186]. Two tyrosine residues, Y63 and Y124, and an arginine, R164, in HCoV-OC43 N-NTD hold the ribonucleotide in place, giving it a particular orientation [186]. However, for SARS-CoV-2 N-NTD, the corresponding residue to HCoV-OC43 N-NTD Y63 is replaced with an alanine, giving a different placement of the UMP and a more spacious binding site [186]. As a result, the HCoV-OC43 N-NTD ribonucleotide binding site is ideal for coordinating the shape of PJ34, and SARS-CoV-2 N-NTD is not, leading to the drug having no effectiveness [186].

## 9. Conclusions and Final Remarks

Undoubtedly, coronaviruses have enormous potential to cause a great deal of harm and mayhem to humans [6,7,9,15,18,130]. Due to the safety precautions necessary to study the more pathogenic coronaviruses, Biosafety Level (BSL) 3 laboratories can be relatively inept, with equipment only providing the most basic requirements [16]. While highly pathogenic coronavirus research is undeniably important in determining antiviral treatment effectiveness before drug approval, endemic coronavirus research provides an opportunity to screen and evaluate antiviral therapeutics and make discoveries in a lower-biosafety-risk manner. Current techniques and methods of endemic coronavirus research in BSL 2 laboratories have finally risen to the challenge [14,23,24,96,168]. Discoveries made in endemic human coronaviruses will likely soar and lead to a deeper understanding of coronavirus biology and host cell biology, as well as better therapeutics.

## Figures and Tables

**Table 2:** Endemic coronavirus model systems with reliable quantification methods and higher viral yields.

	HCoV-OC43	HCoV-NL63	HCoV-229E	HCoV-HKU1	Methodologies Described
**Best Suited Studies**	**Best in studies involving non-structural proteins and genomic replication.**	**Best in studies involving the spike interaction and viral entry.** **Could be utilized to determine the conservation of mechanisms in a different subgenus.**	**Could be utilized to determine the conservation of mechanisms in a different subgenus.**	**Does not cooperate well with isolated cell models; use primary lung epithelial cells [74].**	
Fausto et al., 2020 [14]	VeroE6, 4–5 days post infection (dpi)	LLC-MK2 (6–7 dpi)	Huh7 (3 dpi)		Thorough quantification of HCoV titer by plaque assay, quantification of RNA replication products, and ultracentrifugation for enhanced virus concentration.
Hu et al., 2022 [96]	RD (4.5 dpi), although tested BSC-1, MRC-5	MRC-5 for CPE, and RD for plaque assay (5.5 dpi)	Vero E6 (4 dpi)		Optimized protocol for neutral red staining to determine virus titer by CPE. Plaque assay.
Schirtzinger et al., 2022 [24]	MRC-5 cells produce a higher titer of ~10^7^ plaque-forming units (pfu)/mL and faster (2 dpi) than HRT-18 cells, which produce ~10^6.5^ pfu/mL at 6 dpi. Vero E6 cells also show CPE, but the plaque assay is not ideal.				Improved antibody-based TCID_50_ assay and plaque assay.
Hirose et al., 2021 [23]	Modified Vero E6 cell line with overexpression of TMPRSS2 increases CPE comparatively to both HRT-18 cells and Vero E6. Observations (1–6 dpi).				Confirmed CPE-based TCID_50_ assay with immunoperoxidation assay.
Bracci et al., 2020 [168]	Mv1Lu (5 dpi) MRC-5 (not ideal for plaque assay, although CPE is observed at 4 dpi)	Mv1Lu (5 dpi) MRC-5 (not ideal for plaque assay, although CPE is observed at 4 dpi)			Optimized plaque assay.

Vero E6 is an African green monkey kidney cell line [24], LLC-MK2 is a Rhesus monkey kidney cell line [174], Huh7 is an immortalized human liver cell line [175], RD cells are Rhabdomyosarcoma cells isolated from muscle tissue [176], BSC-1 is an African green monkey kidney cell line [177], MRC-5 are immortalized human lung derived fibroblasts [24], HRT-18 are isolated large intestinal adenocarcinoma cell [24], and Mv1Lu is a mink fetal lung tissue-derived cell line [168].

## Data Availability

The data that support the findings are available in this manuscript.
